# The contribution of the acute phase response to the pathogenesis of relapse in chronic-relapsing experimental autoimmune encephalitis models of multiple sclerosis

**DOI:** 10.1186/s12974-017-0969-4

**Published:** 2017-09-30

**Authors:** Silvy Mardiguian, Emma Ladds, Roberta Turner, Hazel Shepherd, Sandra J. Campbell, Daniel C. Anthony

**Affiliations:** 1 Department of Pharmacology, University of Oxford, Oxford, OX1 4QT UK; 2 Department of Primary Care Health Sciences, University of Oxford, Oxford, OX2 6GG UK

**Keywords:** Multiple sclerosis, Lipopolysaccharide, Acute phase response, VCAM-MPIO

## Abstract

**Background:**

Increased relapse rates in multiple sclerosis (MS) as a consequence of peripheral immune system activation, owing to infection for example, have been widely reported, but the mechanism remains unclear. Acute brain injury models can be exacerbated by augmenting the hepatic acute phase response (APR). Here, we explored the contribution of the hepatic APR to relapse in two rodent models of MS.

**Methods:**

Mice with MOG-CFA-induced chronic relapsing experimental autoimmune encephalitis (CR-EAE) were killed before, during and after the first phase of disease, and the brain and liver chemokine, cytokine and acute phase protein (APP) mRNA expression profile was determined. During remission, the APR was reactivated with an intraperitoneal lipopolysaccharide (LPS) and clinical score was monitored throughout. To explore the downstream mediators, CXCL-1, which is induced as part of the APR, was injected into animals with a focal, cytokine/MOG-induced EAE lesion (fEAE) and the cellularity of the lesions was assessed.

**Results:**

Compared to CFA control, in a rodent CR-EAE model, an hepatic APR preceded clinical signs and central cytokine production in the initial phase of disease. Compared to administration in naïve animals, an LPS challenge during the asymptomatic remission phase of CR-EAE rodents provoked relapse and resulted in the increased and extended expression of specific peripheral hepatic chemokines. CXCL-1 and several other APPs were markedly elevated. A single intravenous administration of the highly induced chemokine, CXCL-1, was found to be sufficient to reactivate the lesions by increasing microglial activation and the recruitment of T cells in fEAE lesions.

**Conclusions:**

The APR plays a contributing role to the pathology seen in models of chronic brain injury and in translating the effects of peripheral immune system stimulation secondary to trauma or infection into central pathology and behavioural signs. Further elucidation of the exact mechanisms in this process will inform development of more effective, selective therapies in MS that, by suppressing the hepatic chemokine response, may prevent relapse.

## Background

The initial coordinated systemic response to physiological challenges such as infection or tissue injury is known as the acute phase response (APR). The APR mobilises the appropriate leukocyte populations to neutralise pathogens, whilst simultaneously initiating repair processes to restore normal function and limit secondary inflammatory damage [1, 2]. The APR occurs via a cascade of local vascular and systemic effects, facilitated by inflammatory mediators including chemokines, cytokines, pentraxins and serum amyloid proteins, which are largely produced by the liver and are often described as acute phase proteins (APP). Most commonly they are defined as a protein whose plasma concentration changes by 25% during an inflammatory response, and they may originate from both hepatic and extra-hepatic sites [2]. Although the APR evolved as a survival mechanism in the short term, prolonged or aberrant effects may be detrimental in chronic inflammation or autoimmune conditions [3].

The introduction of IL-1β into the basal ganglia rapidly induces APP expression by the liver and spleen including chemokine expression such as CXCL1 (the murine equivalent of IL-8) [4]. We have recently shown that astrocyte-shed extracellular vesicles regulate this peripheral APR to inflammatory brain lesions following acute CNS injuries [5] and that augmentation of the peripheral response can exacerbate acute CNS lesions [6]. This appears to be a direct consequence of enhanced mobilisation of leukocytes into the blood by the chemokines released by the liver [7]. Within the brain, multiple sclerosis (MS) lesions are known to be associated with local IL-1β production [8], and MS patients have higher IL-8 serum levels compared to controls [9]. However, it is unknown whether this local cytokine production is able to induce an APR and whether dysregulation of the APR in individuals with MS might impact on pathogenesis of MS. Infections have long been implicated in the pathogenesis in MS, and whilst no single pathogen has been consistently linked to aetiology or relapse, infection would be expected to generate an APR. Indeed, increased relapse rates have been demonstrated in patients with viral [10, 11] and bacterial [12] infections, but the pathway from infection to relapse has not been studied in a systematic manner. In a model of chronic relapsing experimental autoimmune encephalitis (CR-EAE) in SJL mice, Glabinski et al. reported coordinated chemokine upregulation in the brain and spinal cord during clinical relapse, but did not find evidence of CCL2 or CXCL10 expression in the liver [13]. However, in ABH mice with CR-EAE, we discovered that increased hepatic CXCL1 chemokine levels are associated with behavioural depression and the recruitment of neutrophils to the liver. This hepatic neutrophil recruitment was also found to be a histopathological feature in post-mortem liver from MS patients compared to controls [9]. The expression of hepatic CXCL1 in the ABH CR-EAE animals occurred well before the development of overt clinical signs and suggests that activation of the APR might precipitate clinical relapses experienced by MS patients [9]. In another acute EAE model, hepatic CCL2 and CXCL10 expression have been reported to precede the development of fulminant EAE disease, but it was unclear in this study whether this systemic inflammatory response was a ‘primary determinant or a secondary reflection’ of the occurrence of EAE in the affected animals [14].

As for MS, the disease course in EAE models can be affected by systemic cytokine production and infections. Direct intraperitoneal challenge with bacterial enterotoxins [15] or the cytokine IL-12 [16, 17], a critical component of innate resistance to bacterial infection, exacerbates CNS pathology and clinical signs, whilst intraperitoneal inoculation with *Chlamydia pneumonia* [18] or injection of live *Streptococcus pneumonia* [19] worsens disease. Such peripheral physiological challenges have been associated with increased levels of central pro-inflammatory cytokines [20, 21], but the contribution and temporal relationship between infection, the APR and relapse have not been investigated. Microbial recognition induces the production of pro-inflammatory chemokines, necessary for the recruitment of leukocytes to the diseased brain [22]. This has been shown to be important in the pathogenesis of MS and the acute model of EAE [23]. Systemic infection increases peripheral production of chemokines and, on the basis of our finding in acute CNS pathology, might be expected to modulate the pathogenesis of MS. Indeed, peripheral administration of interferon-beta (IFNβ), a mainstay therapy for MS, significantly inhibited hepatic CXCL-1 production and neutrophil recruitment induced by the microinjection of IL-1β into the brain [9].

Should the hepatic APR does play a role in mediating disease exacerbation in response to a peripheral stimulus, the process of recognition and initiation of changes in the chemokine expression profile would necessitate a time lag between the insult and the onset of worsening clinical symptoms. Thus, here we sought to investigate the temporal relationships between a peripheral inflammatory challenge, sufficient to stimulate an APR, and the central and hepatic production of chemokine and APP mRNA compared to the onset and duration of clinical symptoms in chronic models of MS.

## Methods

### Induction of EAE and LPS injection

Chronic relapsing EAE was induced in female Biozzi antibody-high (ABH) mice (6–8 weeks) by subcutaneous injection into both abdominal flanks of 150 μl of an emulsion consisting of 500 μg of mouse spinal cord homogenate in Freund’s incomplete adjuvant (FIA) supplemented with mycobacteria (*Mycobacterium tuberculosis and Mycobacterium butyricum)*. Animals were injected on days 0 and 7 and were weighed daily and assessed for clinical signs according to the following guidelines: 0—healthy, 1—limp tail, 2—incomplete hind limb paralysis, 3—complete hind limb paralysis and 4—forelimb paralysis. Animals (*n* = 6 per group) were killed on days 10, 14, 17 and 28 to examine the APR before, during and after the first phase of disease. A second cohort were established to determine the effect of a lipopolysaccharide (LPS) challenge during the remission period (1 mg/kg *Escherichia coli* 0111:B4; Sigma Chemical Co., St. Louis, MO, USA), when most APPs had returned to baseline. These animals (*n* = 5 or 6 per group) where killed on day 42. All experiments were performed with UK Home Office approval.

### Behavioural testing

Hind limb muscle strength was evaluated using an inverted screen test as previously described [24]. The inverted screen was a square of wire mesh surrounded by a 4-cm-wide wooden edge. A mouse was placed on the screen, which was turned upside down and suspended above soft padding. After 2 min, it was rotated back and the mouse was removed. The time taken for the hind limbs to first drop from the screen, and the time (up to 120 s) that a mouse remained on the screen before falling were recorded. Testing was performed on days 1, 5, 6 and 7 following the lipopolysaccharide (LPS; 1 mg/kg) or vehicle challenge on days 30 and 31. The LPS-treated animals were killed 12 days after the first LPS injection on day 42.

### Induction of focal EAE in Lewis rats

To examine the effect of chemokine administration on the histopathology of an MS-like lesion, focal, cytokine/MOG-induced EAE (fEAE) was generated in male Lewis rats (Charles River, UK) (80–120 g, *n* = 8). Animals were anaesthetized with 1.5–3% isoflurane in a mixture of nitrous oxide/oxygen (70%/30%) and injected subcutaneously at the base of the tail with 100 μl of MOG (35–55) peptide (25 μg diluted in saline) emulsified in IFA (1:1). Control animals were injected with the same volume of saline emulsified in IFA (1:1). To induce the targeted, focal EAE lesions, the immunised animals were anaesthetized with 2–3% isoflurane in a mixture of nitrous oxide/oxygen (70%/30%) and placed in a stereotaxic frame 21 days after the MOG injection (*n* = 8). A midline incision was made in the scalp, and a burr hole, drilled 1 mm anterior and 3 mm lateral to Bregma. Using a finely drawn glass microcapillary (< 50 μm) tip, 2 μl of a cytokine mixture containing 1.45 μg of recombinant rat tumour necrosis factor (TNF; PeproTech London, UK or NIBSC, Potters Bar, UK) and 1 μg of recombinant rat interferon gamma (IFN-γ; PeproTech London, UK) dissolved in sterile saline was injected stereotaxically into the corpus callosum over a 10-min period. After injection, the wound was closed and the animals were allowed to recover from anaesthesia; they displayed no overt clinical signs. A trace of monastral blue (Sigma-Aldrich) was added as a marker dye to enable easier lesion detection histologically. In the absence of an intracerebral cytokine challenge, no MOG-EAE lesions are detectable by MRI or by immunohistochemistry [25]. The focal lesions were allowed to develop and mature for 28 days. We have previously shown that there is no blood-brain barrier breakdown, but that the lesions continue to increase in size. At 28 days after lesion activation, the animals received a single intravenous dose (1 μg) of recombinant rat CXCL-1 (NIBSC, South Mimms) in 0.5 ml endotoxin-free saline or vehicle control.

### EAE tissue collection and immunohistochemistry

The animals were deeply anaesthetised with sodium pentobarbital and transcardially perfused with 0.9% heparinised saline followed by 4% paraformaldehyde in 0.1 M phosphate buffer. Small pieces of fresh liver were removed and frozen immediately in dry ice for PCR analysis. Ten-micrometre-thick sections were cut from the liver and fixed for immunohistochemistry. We have previously shown that the site of the liver biopsy has no effect on the outcome; the expression of proteins is homogenous throughout [26].

Immunohistochemistry was carried out using standard three-step procedures [27] with appropriate biotinylated secondary reagents and standard avidin-biotin complex (ABC) amplification according to the manufacturer’s instructions (Vector Laboratories, Peterborough UK). Neutrophils were identified using an anti-neutrophil serum as previously described [28]. To quantify the neutrophils, four representative, non-overlapping fields were chosen and the average number of positive cells were calculated and expressed per square millimetre.

### fEAE tissue collection

To study the effect of increasing circulating levels of chemokine on the pathogenesis of MS-like disease, we chose to use a focal lesion model where direct histopathological comparison may be made that is not possible in disseminated disease models. Rats with the fEAE were killed 7 days after the injection of CXCL-1 or vehicle, which was the point at which maximum disease activity (clinical signs) was recorded following the LPS challenge in the CR-EAE experiment. Animals were perfusion-fixed with periodate-lysine-paraformaldehyde (PLP) as previously described. Immunohistochemistry was performed to quantify the number of T cells (anti-Phycoerythrin, OX22; Sigma-Aldrich) and activation of microglia (anti-iba1; Abcam, Cambridge, UK). As above, immunohistochemistry was carried out using standard three-step procedures [27] with appropriate biotinylated secondary reagents and standard ABC amplification according to the manufacturer’s instructions (Vector Laboratories, Peterborough UK). The number of iba1 or OX22-postive cells was counted in three non-overlapping high-power (×400) fields in the injected striatum.

### mRNA extraction and RT-PCR

RNA extraction from frozen liver and brain tissues was performed using the Qiagen RNeasy Extraction Kit ® (Qiagen Ltd. Crawley, UK) as per manufacturer’s instructions quantitative real-time polymerase chain reaction (RT-PCR) assays were performed as previously described [29]. Results are expressed as number of copies of target per nanogram input RNA corrected to the housekeeping gene, glyceraldehyde 3-phosphate dehydrogenase (GAPDH).

### Statistical analysis

Data is presented as mean ± SEM at each time point. Independent sample *t* tests were used to compare the behavioural outcomes and motor performance throughout the time course. One-way or two-way ANOVA (Prism 7.0, Graphpad software) was employed for all further analysis.

## Results

### Increased mRNA production of chemokines and APP proteins in the liver precedes clinical signs in EAE

**Fig. 1:**
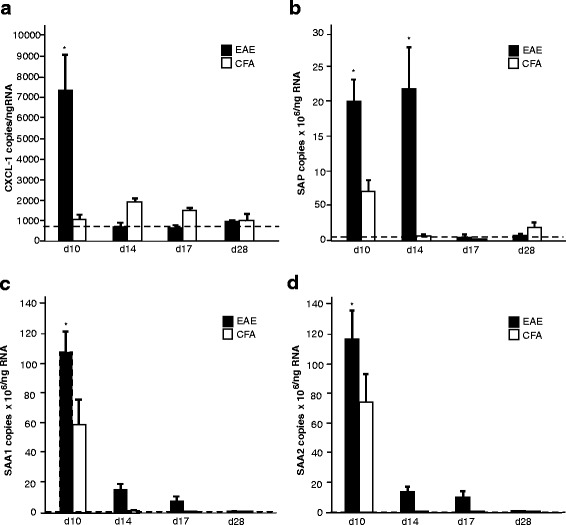
Peripheral production of chemokines and APPs precedes clinical signs in EAE. Quantitative RT-PCR on hepatic tissue collected from EAE animals (*n* = 6) at days 10, 14, 17 and 28 revealed a significantly elevated mRNA expression of CXCL-10 (**a**), SAP (**b**), SAA1 (**c**) and SAA2 (**d**) at day 10, before the onset of clinical signs when compared to CFA mice at the same time point (*p* < 0.05). CXCL-10, SAA1 and SAA2 declined to control levels when the animals were symptomatic (days 14 and 17) and in remission (day 28). SAP levels persisted at day 14 and declined dramatically by day 17. The dotted line indicates the basal level of each marker in naive animals. *p < 0.05

Using quantitative RT-PCR, hepatic tissue collected from EAE and CFA control mice during the pre-symptomatic, symptomatic and remission phases of the disease (Fig. 1) was analysed for chemokine and APP expression. mRNA production of *CXCL1*, *SAA1*, *SAA2* and *SAP* was found to be significantly elevated (*p* < 0.05) in the liver in EAE mice at day 10 (before the onset of clinical signs) compared to levels in both CFA controls and EAE mice during the symptomatic (days 14 and 17) and remission phases of disease (day 28). mRNA *SAP* levels persisted in EAE animals during the symptomatic phase of the disease at day 14, whereas all other expression levels had declined to match those of the controls. By day 17, *SAP* levels had also returned to baseline.

### Increased mRNA production of chemokines and APPs in the CNS in EAE during the acute phase of the disease

Quantitative RT-PCR was used to measure the mRNA production of representative APPs and chemokines in the CNS in tissue collected from EAE and CFA control animals at days 10, 14, 17 and 28 following the induction of EAE.

**Fig. 2:**
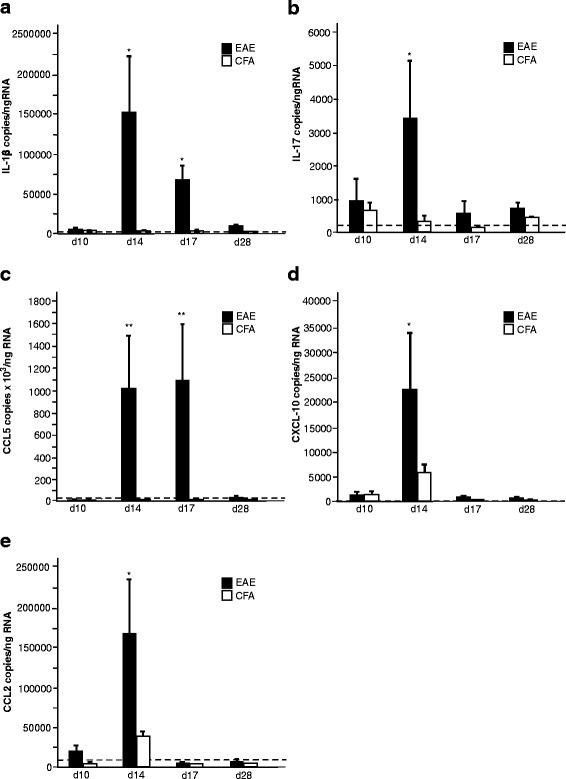
Production of chemokines and APPs in the CNS is highest during the acute phase of EAE. Quantitative RT-PCR on brain tissue collected from EAE and CFA animals (*n* = 6) at days 10, 14, 17 and 28 revealed a significantly elevated mRNA expression of IL-1β (**a**), IL-17 (**b**), CCL5 (**c**) CXCL-10 (**d**) and CCL2 (**e**) at day 14, during the acute phase of the disease when compared to CFA mice (*p* < 0.05) and declined by day 17, with the exception of IL-1β and CCL5 which returned to control levels after day 17. The dotted line indicates the basal level of each marker in naive animals. *p < 0.05

Whilst the production of chemokines and APPs in the periphery was highest at day 10 when the animals were asymptomatic (Fig. 1), in the brain, the highest expression was evident at day 14, during the acute disease phase. Compared to controls, significantly elevated *IL1B* (Fig. 2a) and *CCL5* (*p* < 0.001) (Fig. 2c) intracerebral levels were observed in EAE animals at days 14 and 17 alongside a significant elevation in the expression of *IL17*, *CXCL10* and *CCL2* (*p* < 0.05) at day 14 (Fig. 2).

### Peripheral challenge with LPS exacerbates spontaneous relapse in EAE with a time delay before the onset of clinical symptoms

**Fig. 3:**
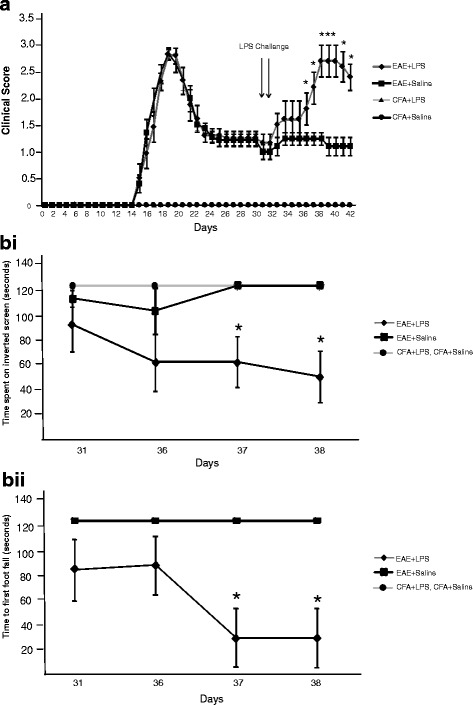
Peripheral LPS administration in EAE exacerbates the relapse phase. EAE mice injected with LPS (*n* = 5) scored more than EAE mice injected with saline (*n* = 5) (**a**) There was a delay of 5 days from the administration of LPS until clinical signs were significantly greater than saline-treated mice. LPS-treated mice performed significantly worse in behavioural tests and spent less time on the inverted screen (**b** (i)) with a reduction in the time of the first foot-fall (**b** (ii)), which achieved significance on days 37 and 38

The first clinical signs were observed in the EAE models at day 13. The disease was most severe at day 17, followed by full or partial remission by day 28. Following the initial acute phase, 30 μg of LPS or saline was injected into EAE mice on days 31 and 32 when the animals were still in remission (Fig. 3a). LPS-treated animals suffered a relapse, with a significant increase in clinical scores from day 36 onwards (*p* < 0.05). There was therefore a time delay of 5 days before significant clinical effects were seen, although a non-significant decline was seen in the intervening period (Fig. 3a). LPS-treated mice also demonstrated a reduction in the period before falling off the inverted screen (Fig. 3b (i)), or before the first double hind limb foot-fault (Fig. 3b (ii)). These results were significant on days 37 and 38 (*p* < 0.05).

### LPS treatment increases peripheral mRNA production of chemokines and APPs in EAE mice but does not affect levels in controls

**Fig. 4:**
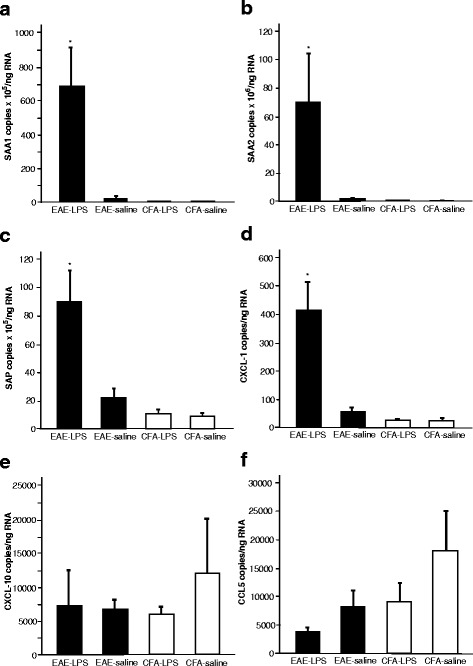
Hepatic mRNA production of acute phase proteins and chemokines increases 8 days after LPS challenge in CR-EAE animals but not in CFA mice. Hepatic mRNA production of SAA1 (**a**), SAA2 (**b**), SAP (**c**) and CXCL-1 (**d**) was significantly higher in LPS-treated EAE mice (*n* = 5) when compared to that in saline-treated EAE mice (*n* = 5) (*p* < 0.05). No significant differences in CXCL-10 (**e**) and CCL5 (**f**) were found between the LPS-challenged mice and saline-treated animals. LPS administration in control CFA mice (*n* = 5) had no effect on the production of these acute phase proteins and chemokines when compared to saline-treated CFA mice (*n* = 4)

The administration of a peripheral LPS challenge in EAE mice at days 31 and 32, i.e. during the disease remission phase, was associated with a significant increase in mRNA expression of the acute phase hepatic proteins *SAA1*, *SAA2*, *SAP* and *CXCL1* (*p* < 0.05) compared to the control mice who received a saline challenge (Fig. 4). LPS challenge was not associated with any altered mRNA expression in control CFA mice, suggesting that the increase was not attributable to the effects of LPS alone, but an interaction of the CR-EAE and the LPS administration. No significant differences were observed in the mRNA production of *CXCL10* or *CCL5*. Neither *IL17* nor tumour necrosis factor (*TNF*) expression was detected in the liver of these animals at this time point (data not shown).

### LPS treatment is associated with increased central mRNA expression of chemokines and APPs in EAE mice compared to controls

**Fig. 5:**
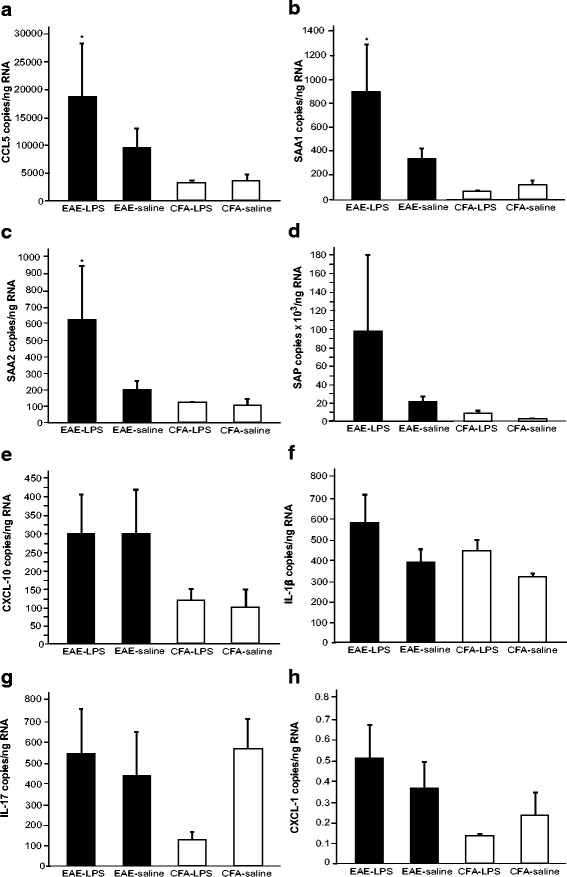
In EAE acute phase, protein and chemokine expression increases in the brain 8 days after LPS challenge without having an effect in CFA mice. mRNA production of CCL5 (**a**), SAA1 (**b**) and SAA2 (**c**) in the brain was higher in LPS-treated EAE mice (*n* = 5) when compared to that in saline-treated EAE mice (*n* = 5) (*p* < 0.05). LPS administration in control CFA mice (*n* = 5) had no effect on the production of these acute phase proteins and expressed levels that were comparable to saline-treated CFA mice (*n* = 4). There were no significant changes in the levels of SAP (**d**), CXCL-10 (**e**), IL-1β (**f**), IL-17 (**g**) and CXCL-1 (**h**)

Seven days after administration of peripheral LPS or saline, mRNA expression of the APPs *SAA1*, *SAA2* and *CCL5* in brain tissue was significantly increased (*p* < 0.05) in LPS-challenged EAE mice (Fig. 5). The expression profiles of *SAP*, *CXCL1*, *CXCL10*, *IL1B* and *IL17* were similar in both cohorts and CFA control mice also demonstrated no significant alterations.

### The peripheral administration of CXCL-1 increases fEAE lesion activity

**Fig. 6:**
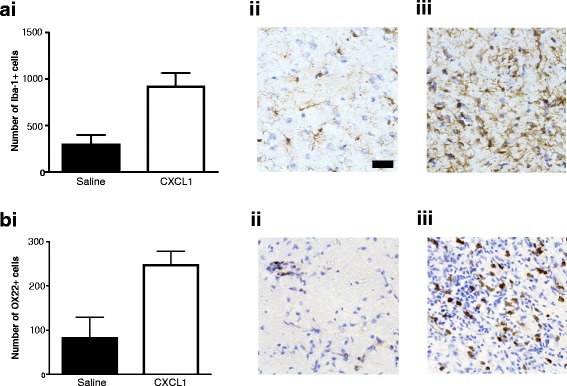
CXCL-1 increases the lesion activity in the fEAE model. At 7 days, animals that received a peripheral injection of 1 μg CXCL-1 iv displayed more (**a**) Iba1 immunoreactive microglia/macrophages (*p* < 0.05) and **b** more T cells (OX-22-positive) were present within the lesions than in those animals that received saline vehicle. Photomicrographs of iba1 (Aii and Aiii) and OX22 (Bii and Biii) staining in the lesions of the control and CXCL-1-treated animals respectively. Scale bar = 20 μm

In order to assess the impact of increased circulating chemokine expression on MS lesions, CR-EAE animals were injected with CXCL-1 (10 μg) protein into the circulation (iv) and the status of inflammatory markers in the lesion tissue assessed using immunohistochemistry. Expression of both Iba-1 and OX-22 was higher 7 days after injection of the chemokine than after a saline injection (*p* < 0.05) (Fig. 6).

## Discussion

Peripheral inflammatory insults, e.g. secondary to peripheral infection [30–32], acute medical illness [33, 34] or the chronic inflammation seen in metabolic syndrome [35] or autoimmune conditions [36], have been associated with an exacerbation of clinical symptoms in a wide range of CNS diseases [37]. Supportive evidence is emerging in many conditions for a mechanistic role played by systematic pro-inflammatory cytokines in this symptomatic deterioration [38–41].

Using the CR-EAE model of MS, in this study, we demonstrate hepatic production of APPs and chemokines prior to the onset of clinical signs. Production was also associated with clinical exacerbation following peripheral inflammatory stimulation during the relapse phase. These results are consistent with a feedforward model of peripheral-central cytokine interaction that underlies both stimulation and perpetuation of the hepatic APR and inflammatory-mediated production of neurological symptoms. Similar extensive cross-talk between peripheral and central insults and the production of central or peripheral cytokines has previously been demonstrated [42, 43]. Both acute and chronic non-immune-mediated injuries to the CNS result in a peripheral APR, which includes expression of hepatic APPs [4, 44]. In this study, acute and chronic non-immune-mediated damage within the CNS is associated with a similar peripheral response to CR-EAE. Therefore, although MS and EAE are primarily considered CNS diseases, the production of peripheral chemokines and APPs may play an important role in the underlying disease process and clinical course.

The presence of a systemic component in MS is not a new idea. Most therapies for MS target processes occurring in the periphery rather than within the CNS. Natalizumab (Tysabri) suppresses peripheral leukocyte migration by both blocking the interaction of VLA4-positive lymphocytes with the brain endothelium and by controlling the localization of marginal zone B cells [45, 46]; fingolimod suppress microglial activation but its principal mode of action is to prevent lymphocyte migration from the lymph nodes [47] and by reducing peripheral secretion of IL-17 by CD8+ T cells [48], whilst glatiramer acetate is thought to compete with myelin antigens within APCs in the periphery although there is very little evidence for this putative mode of action [49]. A more detailed characterisation of the exact processes underlying the peripheral contribution to disease pathology may contribute to the search for additional or more effective therapies.

In this study, expression of peripheral chemokines and APPs reached a peak prior to and then fell at the onset of clinical signs. By contrast, in the CNS, expression was highest during the acute disease phase. It is likely that the cellular source for the APP are astrocytes and microglia as both cell types have been reported to produce these chemokines and cytokines in previous studies [50–52]. Hepatic APP production has previously been shown to increase over a few hours following acute brain injury [4], which was thought to facilitate a concentration gradient firstly between the marrow and the blood and then between the blood and the CNS to encourage central leukocyte migration post-APR stimulation. However, the long-term expression of IL-1β in the brain gave rise to sustained high levels of hepatic cytokines expression [44], suggesting that the postulated role of the cytokines in immune cell recruitment is in fact more complex than the generation of simple gradients and that the continued high level of induction of peripheral cytokines may play a role in more sustained responses.

Here we used LPS, a gram-negative bacterial endotoxin known to cause inflammation [53], to mimic a transient bacterial infection. The peripheral administration of LPS in CR-EAE mice brought forward and exacerbated the clinical signs seen in the disease relapse phase, in keeping with effects seen in other CNS diseases. Importantly, the APR remained elevated for days after the challenge compared the injection of LPS into naïve animals, which highlights the interaction between CNS and peripheral disease, and, once again, suggests that the gradient hypothesis is insufficient to explain the mobilisation and recruitment of leukocytes to the brain. Intraperitoneal LPS administration is also known to exacerbate brain damage and neurological deficits in stroke and prion disease models [38, 54] and is thought to mediate acute cognitive decline via production of pro-inflammatory cytokines [55]. The LPS-induced exacerbation of clinical signs in this study occurred after at least 5 days, longer than the time taken in previous work looking at peripheral LPS challenge [56]. However, this pattern resembles that seen in human MS patients following peripheral infection [11]. The lag may reflect the time needed for sufficient damage to occur to the blood-brain barrier, the destruction of myelin and generation of a conduction block [57].

Whilst MS is thought to largely be a macrophage and T cell-mediated disease, we have previously demonstrated hepatic neutrophil recruitment as a feature of CR-EAE and MS, which appears to reduce during periods of clinical remission [9]. These findings would support an additional role for neutrophils and potentially associated cytokines such as IL-8 and CXCL-1 in the pathological process. As MS patients and CR-EAE models express higher levels of circulating IL-8 and CXCL-1 [58, 59], the hepatic chemokine response may be involved in the control of leukocyte recruitment to chronic inflammatory lesions in the brain. Further support for the involvement of alterations in the peripheral cytokine profile in the pathology contributing to MS comes from studies of natalizumab, which not only inhibits the transmigration of leukocytes into the CNS but also decreases the plasma levels of GM-CSF, IL-6, IL-10 and TNF after 1 year—with IL-8 levels remaining unaltered [46].

In this study, the selective rise in the expression of hepatic chemokines and APPs in CR-EAE mice treated with LPS prior to clinical relapse suggests that LPS is able to induce and maintain the expression of peripheral immune markers. However, the mechanisms for this remain unclear. It has been shown that activation of the innate immune system in MS patients leads to the production of monocyte-derived dendritic cells that can modify autoreactive T cell populations and alter their cytokine secretion profile [60]. We have previously demonstrated that Kupffer cell-depleted CR-EAE rats experience a considerably reduced expression of selected chemokines following central IL-1ß microinjection, highlighting a role for Kupffer cells in the APR. However, this was not associated with decreased neutrophil mobilisation or CXCL-1 and MIP-1ß reduction, indicating that these processes may be Kupffer cell-independent [61]. The induction of high levels of CXCL-1 prompted us to examine the effect of CXCL-1 alone on the pathogenesis of a well-characterised focal model of pattern II MS, which is associated with the deposition of immunoglobulin and complement at sites of active myelin destruction [62]. The formation of stratified lesions in terms of spatial distribution and kinetics distinguishes these models from other types of EAE where lesion presentation is random and variable, and hence is not quantifiable. Here we showed that a single bolus injection of the downstream chemokine CXCL-1 was sufficient to increase microglial activation and increase the presence of T cells within the focal lesions over an extended period. Thus, whilst CXCL-1 is considered to be a neutrophil chemoattractant, it clearly has the potential to exacerbate an antibody-mediated MS-like disease in the CNS, which may be mediated via neutrophil activation or potentially by activating CXCR2 that is known to be expressed on endothelial cells in certain circumstances [63].

## Conclusion

In this study, we demonstrated increased expression of hepatic chemokines and APPs in the CR-EAE model of chronic brain injury. This is consistent with previous findings in models of acute brain injury [4, 9, 44]. Peripheral expression of inflammatory markers was highest whilst the animals were asymptomatic, whereas central chemokine expression was greatest during the acute disease phase, suggesting that peripheral inflammatory mechanisms could contribute to the genesis of central inflammation and subsequent clinical signs. Peripheral LPS challenge results in exacerbation of spontaneous relapse 5 days after LPS administration, and this is associated with an increased expression of hepatic chemokines and APPs. Peripheral administration of CXCL-1 chemokine was sufficient to increase disease activity in focal MS-like lesions and thus chemokine signalling alone can account for the APR-induced exacerbation of central pathology. Therefore, we conclude that the hepatic APR may modulate the impact of the peripheral immune response on central pathology and relapse in MS patients.

## Abbreviations

APPs: Acute phase proteins
CR-EAE: Chronic relapsing experimental autoimmune encephalomyelitis
LPS: Lipopolysaccharide
MRI: Magnetic resonance imaging
MS: Multiple sclerosis
VCAM-1: Vascular cell adhesion molecule-1
